# IMPACT OF COVID-19 MOVEMENT CONTROL ON OLDER ADULTS’ HEALTHCARE UTILIZATION

**DOI:** 10.1093/geroni/igac059.1244

**Published:** 2022-12-20

**Authors:** Vanessa Tan, Gregory Ang, Kim Huat Goh, Adrian Yeow, Gamaliel Tan, Cynthia Chen

**Affiliations:** National University of Singapore, Singapore, Singapore; National University of Singapore, Singapore, Singapore; Nanyang Technological University, Singapore, Singapore; Singapore University of Social Sciences, Singapore, Singapore; Ng Teng Fong General Hospital, Singapore, Singapore; National University of Singapore, Singapore, Singapore

## Abstract

Singapore was one of the first countries affected by COVID-19. Measures to contain the spread of COVID-19 include raising the Disease Outbreak Response System Condition (DORSCON) risk assessment to Orange and instituting a movement control order, termed as the Circuit Breaker. These measures have caused significant disruption in primary care and chronic disease management. As the first point of contact in testing suspected cases, primary care providers shifted their focus from non-COVID-19 services. Using an interrupted time series analysis, we examine the associations of DORSON Orange and Circuit Breaker on acute and chronic primary care visits among older adults aged above 50. We found significant reductions in both acute and chronic primary care visits immediately following DORSCON Orange and Circuit Breaker. DORSCON Orange was associated with a drop of 231 mean acute and chronic daily visits (95% CI -356 to -106). Circuit Breaker was associated with a further drop of 268 mean daily visits (95% CI -426 to -111). These reductions were also observed for acute and chronic visits separately. Routine chronic care appointments were deferred or cancelled to reduce the risk of transmission as patients with underlying medical conditions were at higher risk of developing severe complications. Delayed access to primary care can have profound health implications, especially for older adults. Ensuring accessibility to primary care is a key priority in maintaining population health. Understanding the impact of COVID-19 tightening measures on older adults’ primary care utilisation will be useful for future public health planning.

